# Synthesis of Some New 1,3,4-Thiadiazole, Thiazole and Pyridine Derivatives Containing 1,2,3-Triazole Moiety

**DOI:** 10.3390/molecules22020268

**Published:** 2017-02-10

**Authors:** Nadia A. Abdelriheem, Ali M. M. Mohamed, Abdou O. Abdelhamid

**Affiliations:** Department of Chemistry, Faculty of Science, Cairo University, Giza 12613, Egypt; Nadia.abdelhamid5@gmail.com (N.A.A.); Ali.egypt3@gmail.com (A.M.M.M.)

**Keywords:** 1,3,4-thiadiazoles, 1,2,3-triazoles, hydrazonoyl halides, pyridines, nicotinic ester

## Abstract

In this study, 1-(5-Methyl-1-(*p*-tolyl)-1*H*-1,2,3-triazol-4-yl)ethan-1-one, was reacted with Thiosemicarbazide, alkyl carbodithioate and benzaldehyde to give thiosemicarbazone, alkylidenehydrazinecarbodithioate and 3-phenylprop-2-en-1-one-1,2,3-triazole derivatives. The 1,3,4-thiadiazole derivatives containing the 1,2,3-triazole moiety were obtained via reaction of alkylidenecarbodithioate with hydrazonoyl halides. Also, hydrazonoyl halides were reacted with thiosemicarbazone and pyrazolylthioamide to give 1,3-thiazoles derivatives. Subsequently, 3-phenyl-2-en-1-one was used to synthesize substituted pyridines and substituted nicotinic acid ester. The latter was converted to its azide compound which was reacted with aromatic amines and phenol to give substituted urea and phenylcarbamate containing 1,2,3-triazole moiety. The newly synthesized compounds were established by elemental analysis, spectral data and alternative synthesis whenever possible.

## 1. Introduction

In synthesis, 1,2,3-triazoles are useful building blocks and are additionally important due to their broad range of biological activities [1,2]—they are stable to moisture, oxygen, light and metabolic process. A series of novel 1,2,3-triazoles were synthesized [3] and found to have cytotoxic activity against human cancer cell lines such as U937, THP-1, HL60 and B16-F10. The 1,3,4-thiadiazole ring is one of the most important and well-known heterocyclic nuclei, as a common and integral feature of a variety of natural products and medicinal agents. As a core structural component, 1,2,4-thiadiazole is present in an array of drug categories such as antimicrobial, anti-inflammatory, analgesic, antiepileptic, antiviral, antineoplastic, antitubercular and antinociceptive agents [4,5]. Thiazoles display a broad range of biological activities and are found in many potent biologically active molecules such as antimicrobial, antifungal and antineoplastic drugs [6]. However, they are mostly known for their anticancer [7] and antimicrobial [8] activities. Also, pyridine derivatives, including those bearing various heterocyclic nuclei, have shown potent pharmacological properties, including antifungal [9,10], antitubercular [11], antimalarial [12], antibacterial [13], antimicrobial [14], or insecticide [15]. We report here the synthesis of new 1,3,4-thiadiazoles, 5-arylazothiazoles, and pyridines containing 1,2,3-triazole moiety.

## 2. Results

**Scheme 1:**
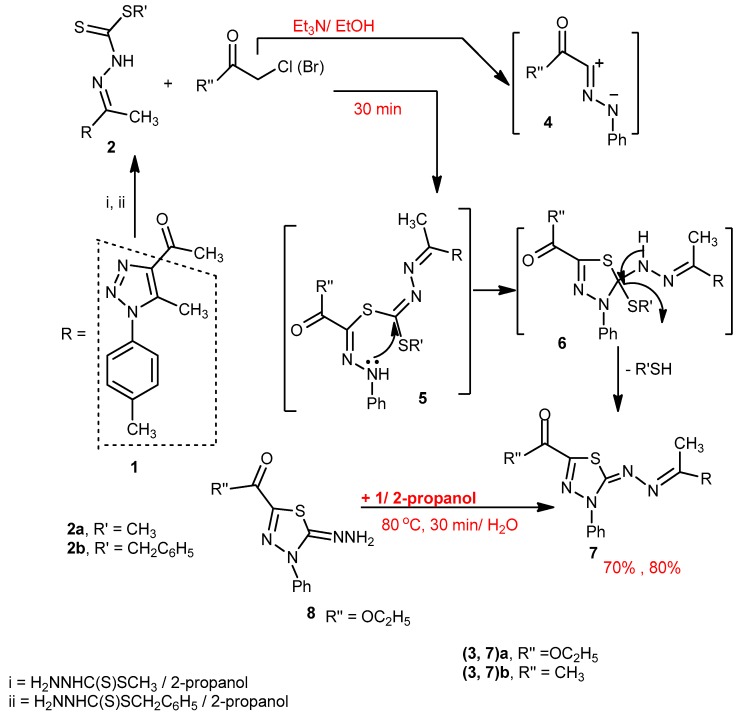
Synthesis of 1,3,4-thiadiazoles **7a**,**b**.

Treatment of 1-(5-methyl-1-(*p*-tolyl)-1*H*-1,2,3-triazol-4-yl)ethan-1-one (**1**) [16] with methyl or benzyl carbodithioate [16,17] in 2-propanol gave the corresponding methyls 2-(1-(5-methyl-1-(*p*-tolyl)-1*H*-1,2,3-triazol-4-yl)ethylidene)hydrazinecarbodithioate (**2a**) [17] and benzyl 2-(1-(5-methyl-1-(*p*-tolyl)-1*H*-1,2,3-triazol-4-yl)ethylidene)hydrazinecarbodithioate (**2b**) [18], respectively (Scheme 1). Structures **2a** and **2b** were elucidated by elemental analyses, spectral data and chemical transformation. Thus, treatment of **2a** or** 2b** with ethyl 2-chloro-2-(2-phenylhydrazono)acetate (**3a**) in ethanolic triethylamine at room temperature gave one isolated product formulated as ethyl 5-((1-(5-methyl-1-(*p*-tolyl)-1*H*-1,2,3-triazol-4-yl)ethylidene)-hydrazono)-4-phenyl-4,5-dihydro-1,3,4-thiadiazole-2-carboxylate (**7a**) (Scheme 1). The latter was confirmed by elemental analysis, spectral data, and an alternative synthesis route. Thus, ethyl 5-hydrazono-4-phenyl-4,5-dihydro-1,3,4-thiadiazole-2-carboxylate (**8**) [19] was reacted with compound **1**, in 2-propanol to give a product identical in all aspects (m.p., mixed m.p., and spectra) with **7a**.

In light of these results, the mechanism outlined in Scheme 1 seems to be the most plausible pathway for the formation of **7a **from the reaction of the **2a **(or** 2b**) with **3a**. The reaction involves initial formation of thiohydrazonate **5**, which undergoes intermolecular cyclization as soon as it is formed to yield the intermediate **6 **or via 1,3-dipolar cycloaddition of nitrilimine **4a **(generated in situ from **3a **with triethylamine) to the C=S double bond of **2**. The formations of **5 **and **6 **are similar to the reactions of hydrazonoyl halides with 1-phenyl-1,4-dihydrotetrazole-5-thione [20] and 5-phenyl-1,3,4-thiadiazole-2(3*H*)-thione [21]. Intermediate **6 **was converted to **7 **by elimination of methanthiol (or benzylthiol). Analogously, treatment of the appropriate **2a **(or** 2b**) with **3b** gave 2,3-dihydro-1,3,4-thiadiazoles **7b**, in good yield (Scheme 1).

**Scheme 2:**
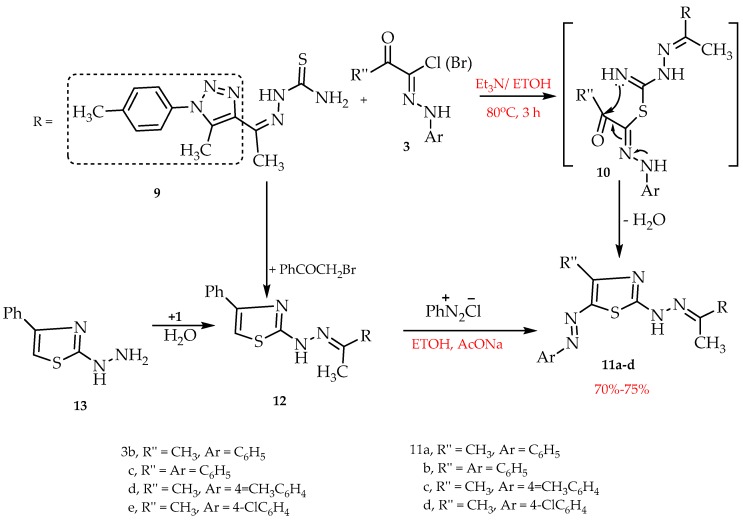
Synthesis of thiazoles **11a**–**d**.

After 2-(1-(5-Methyl-1-(*p*-tolyl)-1*H*-1,2,3-triazol-4-yl)ethylidene)hydrazinecarbothioamide (**9**) [21] was reacted with hydrazonyl chloride **3c **in ethanolic triethylamine under reflux to give the corresponding(2-(2-(1-(5-methyl-1-(*p*-tolyl)-1*H*-1,2,3-triazol-4-yl)ethylidene)-hydrazinyl)-4-phenyl-5-(phenyldiazenyl)thiazole (**11b**) in quantitative yield (Scheme 2), structure **11b **was confirmed by elemental analysis, spectral data and alternative synthesis. Thus, 2-(2-(1-(5-methyl-1-(*p*-tolyl)-1*H*-1,2,3-triazol-4-yl)ethylidene)-hydrazinyl)-4-phenylthiazole (**12**) [22], prepared from reaction of **1 **with 2-hydrazinyl-4-phenylthiazole (**13**) [23], or reaction of **9** with ω-bromoacetophenone [21], was coupled with benzenediazonium chloride in ethanolic sodium acetate at 0–5 °C to furnish a product identical in all aspects (m.p., mixed m.p., and spectra) to **11b**. Analogously, treatment of **9 **with the appropriate **3b**,**d**,**e **gave thiazole derivatives **11a**,**c**,**d** respectively, in good yields (Scheme 2).

**Scheme 3:**
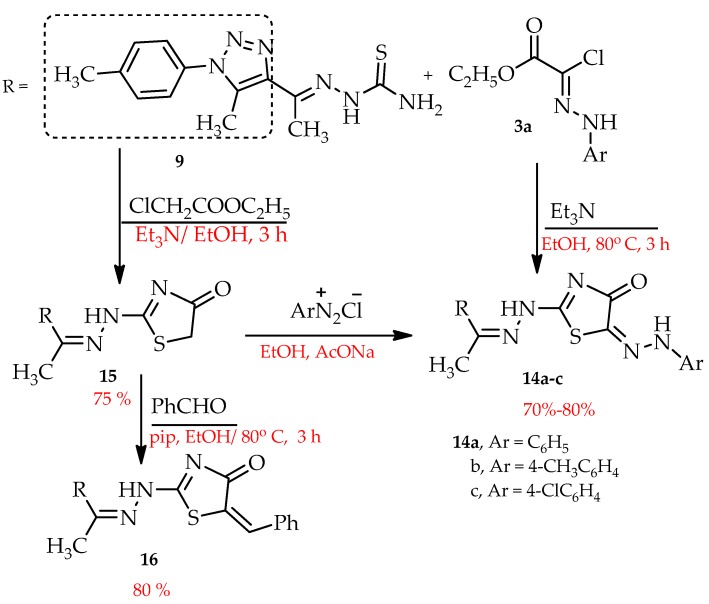
Synthesis of thiazolone **14a**–**d**.

A similar treatment of **9 **with ethyl 2-chloro-2-(2-phenylhydrazono)acetate (**3a**) in ethanolic triethylamine gave 2-(2-(1-(5-methyl-1-(*p*-tolyl)-1*H*-1,2,3-triazol-4-yl)ethylidene)-hydrazinyl)-5-(2-phenylhydrazono)thiazol-4(5*H*)-one (**14a**) (Scheme 3). Structure **14a** was elucidated by elemental analysis, spectral data and an alternative synthetic route. Thus, treatment of benzenediazonium chloride with 2-(2-(1-(5-methyl-1-(*p*-tolyl)-1*H*-1,2,3-triazol-4-yl)ethylidene)-hydrazinyl)thiazol-4(5*H*)-one (**15**), prepared via reaction of **9** with ethyl chloroacetate in boiling ethanol, in a cold ethanolic sodium acetate solution, afforded a product identical in all aspects (m.p., mixed m.p., and spectra) with **14a**. 

Analogously, the appropriate arenediazonium chloride was coupled with **15** in ethanolic sodium acetate afforded (2-(2-(1-(5-methyl-1-(*p*-tolyl)-1*H*-1,2,3-triazol-4-yl)ethylidene)-hydrazinyl)-5-(2-arylhydrazono)thiazol-4(5*H*)-one **14b **and** 14c**; respectively (Scheme 3). Also, compound **15** was reacted with benzaldehyde in ethanol in the presence of a catalytic amount of piperidene, giving 5-(benzylidene)-2-(2-(1-(5-methyl-1-(*p*-tolyl)-1*H*-1,2,3-triazol-4-yl)ethylidene)-hydrazinyl)thiazol-4(5*H*)-one (**16**).

**Scheme 4:**
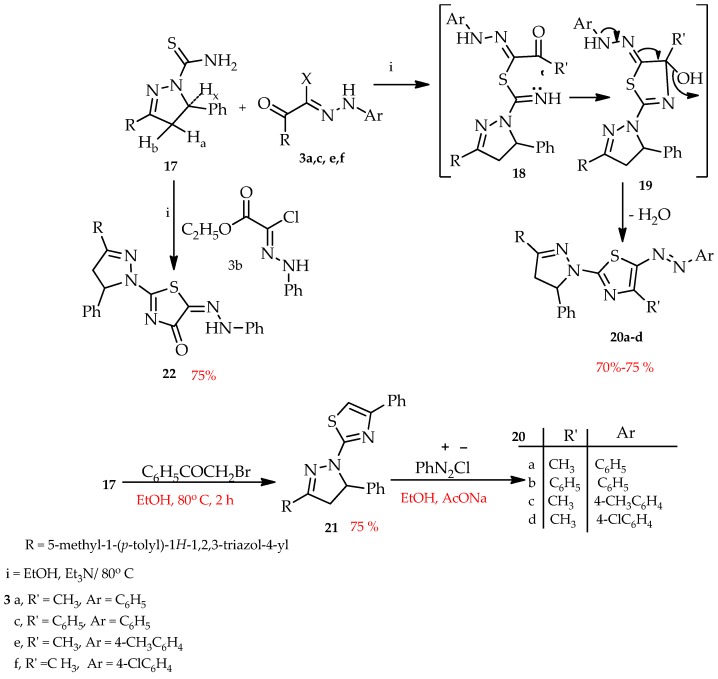
The 2-(3-(5-Methyl-1-(*p*-tolyl)-1*H*-1,2,3-triazol-4-yl)-5-phenyl-4,5-dihydro-1*H*-pyrazol-1-yl)-5-(aryldiazenyl)-4-substituted thiazole 20**a**–**d**.

Treatment of 3-(5-methyl-1-(*p*-tolyl)-1*H*-1,2,3-triazol-4-yl)-5-phenyl-4,5-dihydro-1*H*-pyrazole-1-carbothioamide (**17**) [24,25] with the appropriate α-keto-hydrazonoyl halides **3a**,**c**,**e**,**f **in ethanolic triethylamine afforded 5-(aryldiazenyl)-4-substituted-2-(3-(5-methyl-1-(*p*-tolyl)-1*H*-1,2,3-triazol-4-yl)-5-phenyl-4,5-dihydro-1*H*-1*H*-pyrazol-1-yl)-5-(aryldiazenyl)-4-substituted thiazole **20a**–**d**, respectively (Scheme 4). Structures **20a**–**d** were elucidated via elemental analyses, spectral data and alternative synthetic routes. Thus, 2-(3-(5-methyl-1-(*p*-tolyl)-1*H*-1,2,3-triazol-4-yl)-5-phenyl-4,5-dihydro-1*H*-pyrazol-1-yl)-4-phenylthiazole (**21**) was coupled with benzenediazonium chloride in ethanolic sodium acetate solution at 0–5 °C, affording a product identical in all aspects (m.p., mixed m.p., and spectra) with **20b**.

In the light of these results, the mechanism outlined in Scheme 4 seems to be the most plausible pathway for the formation of **20 **from the reaction of **17 **with **3**. The reaction involves initial formation of thiohydrazonate **18**, which undergoes cyclization as soon as it is formed to yield the intermediate **19**. The latter suffers dehydration to the final product **20.**

Treatment of **17** with **3b** in ethanolic triethylamine gave 2-(3-(5-methyl-1-(*p*-tolyl)-1*H*-1,2,3-triazol-4-yl)-5-phenyl-4,5-dihydro-1*H*-pyrazol-1-yl)-5-(2-phenylhydrazono)thiazol-4(5*H*)-one (**22**) in a good yield. Structure **22 **was confirmed by elemental analysis and spectral data.

**Scheme 5:**
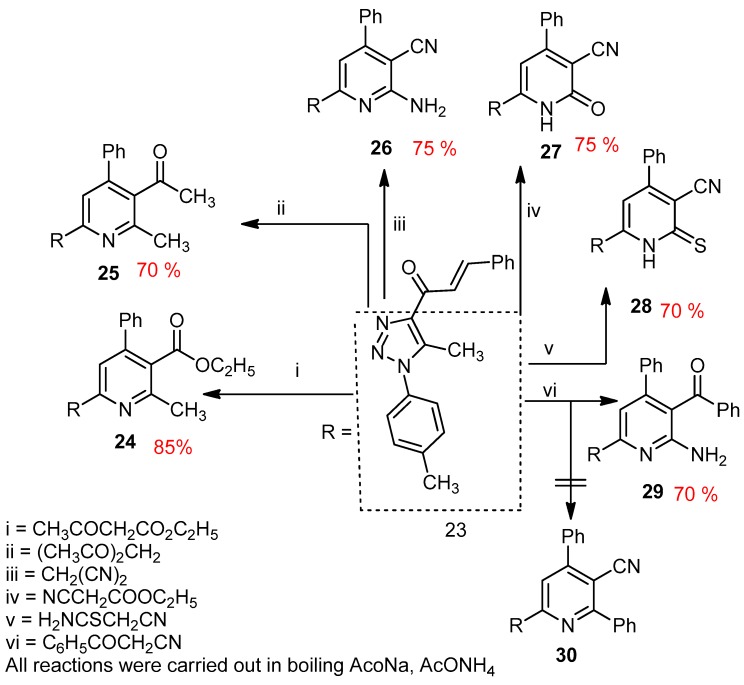
Synthesis of substituted pyridine derivatives **24**–**29**.

Next, treatment of compound **23** with each of ethyl acetoacetate, acetylacetone, malononitrile, ethyl cyanoacetate, cyanothioacetamide and benzoylacetonitrile in acetic acid containing ammonium acetate afforded pyridine derivatives **24**–**29**, respectively (Scheme 5). Structures **24**–**29 **were elucidated on the basis of elemental analysis, spectral data and chemical transformation (cf. Experimental and Scheme 5). ^1^H-NMR spectrum of **24** showed signals at δ = 1.34 (t, 3H, **CH_3_**CH_2_O), 2.4 (s, 3H, 4-CH_3_C_6_H_4_), 2.60 (s, 3H, CH_3_, pyridine H-2), 2.69 (s, 3H, CH_3_, triazole H-5), 4.2 (q, 2H, CH_3_**CH_2_**O), 7.27–7.73 (m, 9H, ArH’s), 7.90 (s, 1H, pyridine H-5). 

**Scheme 6:**
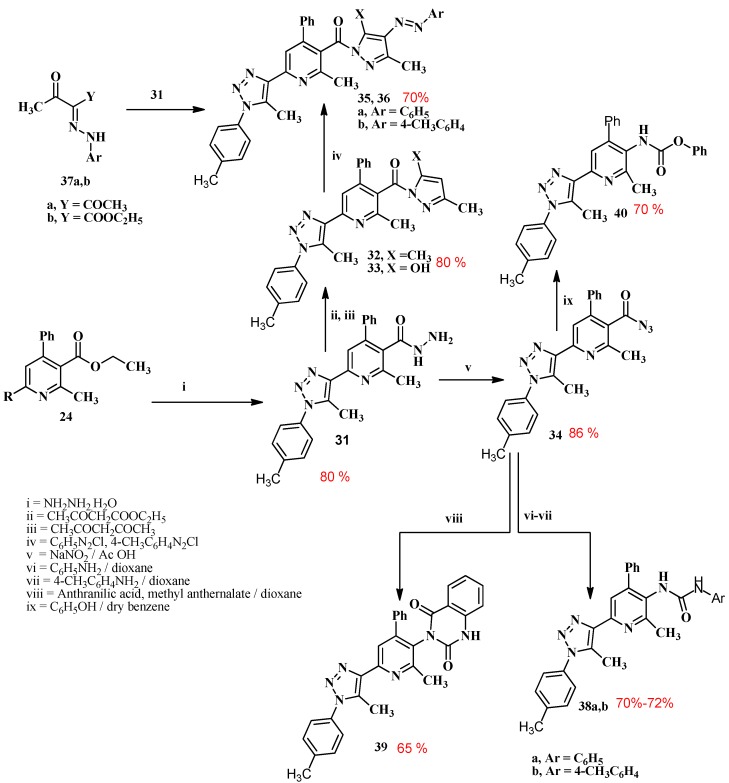
Synthesis of pyrazoles, urea, quinazoline and carbamate.

Thus, treatment of **24** with hydrazine hydrate in boiling ethanol gave 2-methyl-6-(5-methyl-1-(*p*-tolyl)-1*H*-1,2,3-triazol-4-yl)-4-phenylnicotinohydrazide (**31**) in a good yield. Structure **31** was elucidated via elemental analyses, spectral data and chemical transformation. Compound **31 **was reacted with each of acetylacetone, ethyl acetoacetate, or with sodium nitrite in the presence of acetic acid to give **32**, **33** and azido **34**, respectively (Scheme 6).

Meanwhile, each of the compounds **32 **and **33 **were reacted with benzenediazonium chloride in ethanolic sodium acetate solution, giving (3,5-dimethyl-4-(phenyldiazenyl)-1*H*-pyrazol-1-yl)-(2-methyl-6-(5-methyl-1-(*p*-tolyl)-1*H*-1,2,3-triazol-4-yl)-4-phenylpyridin-3-yl)methanone (**35a**) and 5-methyl-2-[2-methyl-6-(5-methyl-1-(p-tolyl)-1H-[1,2,3]triazol-4-yl)-4-phenylpyridine-3-carbonyl]-4-(phenyl-hydrazono)-2,4-dihydro-pyrazol-3-one (**36a**) (Scheme 6). The structure of compounds **35a **and **36a **were confirmed by alternative synthesis, by treatment of the hydrazide **31 **with each of 3-(2-phenylhydrazono)pentane-2,4-dione (**37a**) [26] and ethyl 3-oxo-2-(phenylhydrazono)butanoate (**37b**) [27] in boiling acetic acid for products identical in all aspects (m.p., mixed m.p., and spectra) with **35a **and **36a**, respectively**. **

Analogously, *p*-tolyldiazonium chloride was reacted with each **32 ** and **33**, giving (3,5-dimethyl-4-(*p*-tolyldiazenyl)-1*H*-pyrazol-1-yl)(2-methyl-6-(5-methyl-1-(*p*-tolyl)-1*H*-1,2,3-triazol-4-yl)-4-phenylpyridin-3-yl)methanone (**35b**) and 5-methyl-2-(2-methyl-6-(5-methyl-1-(*p*-tolyl)-1*H*-1,2,3-triazol-4-yl)-4-phenylnicotinoyl)-4-(2-(*p*-tolyl)-hydrazono)-2,4-dihydro-3*H*-pyrazol-3-one (**36b**), respectively (Scheme 6).

Azido(2-methyl-6-(5-methyl-1-(*p*-tolyl)-1*H*-1,2,3-triazol-4-yl)-4-phenylpyridin-3-yl)methanone (**34**) can be converted into urea derivatives, **38a**,**b** and 3-(2-methyl-6-(5-methyl-1-(*p*-tolyl)-1*H*-1,2,3-triazol-4-yl)-4-phenylpyridin-3-yl)quinazoline-2,4(1*H*,3*H*)-dione (**39**) by being boiled with the appropriate aromatic amines, or anthranilic acid in dry dioxane, respectively. Also, phenyl 2-methyl-6-(5-methyl-1-(*p*-tolyl)-1*H*-1,2,3-triazol-4-yl)-4-phenylpyridin-3-ylcarbamate **40 **can be obtained by boiling the azido **34 **with phenol in dry benzene (Scheme 6). 

## 3. Materials and Methods 

All meeting points were determined on an electro thermal Gallen Kamp melting point apparatus (Laim George, Calgary, AB, Canada) and are uncorrected. IR (cm^−1^) spectra were recorded on KBr disk on a FTIR-8201 spectrophotometer (Shimadzu, Tokyo, Japan). ^1^H-NMR and ^13^C-NMR spectra were measured in deuterated dimethyl sulfoxide (DMSO-*d*6) using a Varian Gemini 300 NMR spectrometer (Varian, Inc., Karlsruhe, Germany). Mass spectra were recorded on a Shimadzu GCMS-QP1000 EX mass spectrometer (Tokyo, Japan) at 70 eV. Measurements of the elemental analysis were carried out at the Microanalytical Centre of Cairo University, Giza, Egypt. All reactions were followed by TLC (Silica gel, Merck, Kenilworth, NJ, USA). Hydrazonoyl halides were prepared as previously reported [28,29,30,31]

### 3.1. Alkyl 2-(1-(5-Methyl-1-(p-tolyl)-1H-1,2,3-triazol-4-yl)-ethylidene)hydrazine-1-carbodithioate ***2a*** and ***2b***

A mixture of 1-(5-methyl-1-(*p*-tolyl)-1*H*-1,2,3-triazol-4yl)ethanone (**1**) [16] (1 g, 5 mmol) and alkyl carbodithioate (5 mmol) in 2-propanol (20 mL) was refluxed for 30 min. The reaction mixture was cooled and the resulting solid was collected and crystallized from the proper solvent to give **2a**,**b**.

*Methyl 2-(1-(5-methyl-1-(p-tolyl)-1H-1,2,3-triazol-4-yl)-ethylidene)hydrazine-1-carbodithioate* (**2a**). Buff crystals from ethanol: yield: 75% , m.p.: 186 °C, FT-IR (KBr, cm^−1^): 3522 (NH), 3064 (CH), 1603 (C=N), 1561 (C=C); ^1^H-NMR (300 MHz, DMSO-*d*_6_): δ = 2.36 (s, 3H, CH_3_), 2.45 (s, 3H, CH_3_), 2.50 (s, 3H, CH_3_), 3.20 (s, 3H, CH_3_), 7.38–7.51 (m, 4H, ArH^’^s) and 12.4 (s, br, 1H, NH). Anal. Calcd. For C_14_H_17_N_5_S_2_ (319.46) C, 52.64; H, 5.36; N, 21.92; S, 20.07 Found C, 52.70; H, 5.40; N, 21.90; S, 20.18.

*Benzyl 2-(1-(5-methy-1-(p-tolyl)-1H-1,2,3-triazol-4-yl)-ethylidene)hydrazine-1-carbodithioate* (**2b**). Buff crystals from DMF: yield 75%, m.p.: 324 °C, FT-IR (KBr, cm^−1^): 3421 (NH), 3052 (CH), 1611 (C=N), 1553 (C=C); ^1^H-NMR (300 MHz, DMSO-*d*_6_): δ = 2.41 (s, 3H, CH_3_), 2.50 (s, 3H, CH_3_), 2.73 (s, 3H, CH_3_), 3.28 (s, 2H, CH_2_), 7.39–7.47 (m, 9H, ArH’s) and 12.35 (s, br, 1H, NH). Anal. Calcd. For C_20_H_21_N_5_S_2 _(395.54) C, 60.73; H, 5.35; N, 17.71; S, 16.21 Found C, 60.69; H, 5.32; N, 17.68; S, 16.30. 

### 3.2. 5-((1-(5-Methyl-1-(p-tolyl)-1H-1,2,3-triazol-4-yl)ethylidene-4-phenyl-4,5-dihydro-1,3,4-thiadiazole-derivatives ***7a***,***b***

**Method A: **Triethyl amine (0.75 mL, 0.5 g, 5 mmol) was added dropwise with stirring to a mixture of the appropriate alkyl carbodithioate **2a **or** 2b **(5 mmol) and the appropriate hydrazonoyl halides **3a**,**b **[27,28,29,30] (5 mmol) in ethanol (20 mL). The resulting solid which formed after 30 min was collected and crystallized from the proper solvent to give the corresponding thiadiazole derivatives **7a**,**b**.

*Ethyl 5-((-1-(5-methyl-1-(p-tolyl)-1H-1,2,3-triazol-4-yl)ethylidene)hydrazono)-4-phenyl-4,5-dihydro-1,3,4-thiadiazole-2-carboxylate* (**7a**). Yellow crystals from acetic acid Yield: 70%, m.p.: 205–207 °C; FT-IR (KBr, cm^−1^): 3047 (CH), 1708 (CO), 1616 (C=N), 1573 (C=C) ; ^1^H-NMR (300 MHz, CDCl_3_): δ = 1.59 (t, 3H, CH_2_-**CH_3_**), 2.48 (s, 3H, CH_3_), 2.64 (s, 3H, CH_3_), 2.71 (s, 3H, CH_3_), 4.50 (q, 2H, **CH_2_**-CH_3_), and 7.26–8.18 (m, 9H, AH’s); MS (El, *m*/*z *(%)): 461 (M^+^,100), 433 (20), 400 (80), 289 (20), 243 (20), 184 (30), 91 (30), 80 (100), 64 (40); Anal. Calcd. For C_23_H_23_N_7_SO_2_ (461.55), C, 59.85; H, 5.02; N, 21.24; S, 6.95 Found C, 59.90; H, 5.12; N, 21.34; S, 6.99

*1(5-((1-(5-Methyl-1-(p-tolyl)-1H-1,2,3-triazol-4-yl)ethylidene)hydrazono)-4-phenyl-4,5-dihydro-1,3,4-thiadiazol-2-yl)ethan-1-one* (**7b**). Yellow crystals from ethanol. Yield: 80%, m.p.: 270–271 °C; FT-IR (KBr, cm^−1^): 2924 (CH),1678 (CO), 1616 (C=N), 1573 (C=C); ^1^H-NMR (300 MHz, CDCl_3_): δ = 2.48 (s, 3H, CH_3_), 2.63 (s, 3H, CH_3_), 2.65 (s, 3H, CH_3_), 2.71 (s, 3H, CH_3_), and 7.26–8.14 (m, 9H, ArH’s); ^13^C-NMR in CHCl_3_ δ = 9.4 (5-CH_3_ triazole), 13.9 (=CH_3_), 19.9 (4-CH_3_C_6_H_4_), 24.7 (CH_3_CO), 123.3, 125.4, 127.2, 127.8, 129.3, 130.2, 132.4, 139.7, 140.7, 142.33, 147.8, 152.7, 163.8, 189.1 (CO), MS (El, *m*/*z *(%)): 431 (M^+^, 100), 403 (5), 370 (10), 360 (30), 301 (10), 259 (15), 194 (55), 184 (3), 172 (40), 91 (50), 80 (100), 64 (50); Anal. Calcd. For C_22_H_21_N_7_OS (431.52), C, 61.23; H, 4.91; N, 22.72; S, 7.43 Found C, 61.40; H, 4.89; N, 22.80; S, 7.80

Alternative synthesis of Ethyl 5-(1-(5-methyl-1-(*p*-tolyl)-1*H*-1,2,3-triazol-4-yl)ethylidene-4-phenyl-4,5-dihydro-1,3,4-thiadiazole-2-carboxylate (**7a**).

**Method B:** A mixture of ethyl 5-hydrazono-4-phenyl-4,5-dihydro-1,3,4-thiadiazole-2-carboxylate (**8**) [18] (1.3 g, 5 mmol) and 1-(5-methyl-1-(*p*-tolyl)-1*H*-1,2,3-triazol-4-yl)ethanone (**1**) (1 g, 5 mmol) in 2-propanol were heated for 30 min. The crude solid that was collected and crystallized from ethanol gave a product identical in all aspects (m.p., mixed m.p. and spectra) with **7a**.

### 3.3. 2-(2-(1-(5-Methyl-1-(p-tolyl)-1H-1,2,3-triazol-4-yl)ethylidene-hydrazinyl-thiazole derivatives ***11a***–***d***

**Method A**: A mixture of **9** (1.4 g, 5 mmol), the appropriate hydrazonoyl halides **3b**–**e** (5 mmol) and triethylamine (0.5 g, 0.7 mL, 5 mmol) in ethanol was heated under reflux for 3 h. The resulting solid that was collected and recrystallized gave thiazole derivatives **11a**–**d.**

*4-Methyl-2-(2-((E)-1-(5-methyl-1-(p-tolyl)-1H-1,2,3-triazol-4-yl)ethylidene)hydrazinyl)-5-((E)-phenyldiazenyl)thiazole* (**11a**). Orange crystals from acetic acid; Yield: 75%, m.p.: 255 °C; FT-IR (KBr, cm^−1^): 3417 (NH), 3032 (CH), 1600 (C=C); ^1^H-NMR (300 MHz, CDCl_3_): δ = 2.48 (s, 3H, CH_3_), 2.59 (s, 3H, CH_3_), 2.65 (s, 3H, CH_3_), 3.3 (s, 3H, CH_3_), 6.96–7.55 (m, 9H, ArH’s) and 9.18 (s, br, 1H, NH). ^13^C-NMR (DMSO-*d*_6_) δ = 8.1, 12.9, 13.7, 20.8, 114.4, 121.6, 123.8, 129.0, 129.6, 129.9, 130.4, 139.1, 139.6, 146.2, 154.6, 160.1, 164.9. Anal. Calcd. For C_22_H_22_N_8_S (430.54): C, 61.37; H, 5.15; N, 26.03; S, 7.4 Found C, 61.40; H, 5.10; N, 26.13; S, 7.50.

*2-(2-((E)-1-(5-Methyl-1-(p-tolyl)-1H-1,2,3-triazol-4-yl)ethylidene)hydrazinyl)-4-phenyl-5-((E)-phenyldiazenyl)thiazole* (**11b**). Orange crystals from ethanol, Yield: 70%, m.p.: 245 °C; FT-IR (KBr, cm^−1^): 3417 (NH), 3074 (CH), 1578 (C=C); ^1^H-NMR (300 MHz, DMSO-*d*_6_): δ = 2.45 (s, 3H, CH_3_), 2.49 (s, 3H, CH_3_), 3.30 (s, 3H, CH_3_), 7.32–8.28 (m, 14H, ArH’s) and 10.71 (s, br, 1H, NH). Anal. Calcd. For C_27_H_24_N_8_S (492.61): C, 65.83; H, 4.91; N, 22.75; S, 6.51; Found C, 65.79, N, 22.78, S, 6.561.

*4-Methyl-2-(2-((E)-1-(5-methyl-1-(p-tolyl)-1H-1,2,3-triazol-4-yl)ethylidene)hydrazinyl)-5-((Z)-p-tolyldiazenyl)thiazole* (**11c**). Gray crystals from acetic acid, Yield: 70%, m.p.: 250 °C; FT-IR (KBr, cm^−1^): 3421 (NH), 3020 (CH), 1593 (C=C); ^1^H-NMR (300 MHz, DMSO-*d*_6_): δ = 2.30 (s, 3H, CH_3_), 2.44 (s, 3H, CH_3_), 2.58 (s, 3H, CH_3_), 2.65 (s, 3H, CH_3_), 2.75 (s, 3H, CH_3_), 7.15–7.53 (m, 8H, ArH’s), 10.54 (s, br, 1H, NH). ^13^C-NMR (DMSO-*d*_6_) δ = 8.1, 12.9, 13.7, 20.8, 21.5, 114.2, 122.0, 123.8, 129.0, 129.6, 129.9, 136.8, 139.4, 139.8, 146.8, 151.6, 156.8, 164.7. Anal. Calcd. For C_23_H_24_N_8_S (444.57), C, 62.14; H, 5.44; N, 25.21; S, 7.21 Found C, 62.15; H, 5.55; N, 25.25; S, 7.30. 

*5-((Z)-(4-Chlorophenyl)diazenyl)-4-methyl-2-(2-((E)-1-(5-methyl-1-(p-tolyl)-1H-1,2,3-triazol-4-yl)ethylidene)hydrazinyl)thiazole* (**11d**). Red crystals from ethanol, Yield: 70%, m.p.: 240 °C; FT-IR (KBr, cm^−1^): 3387 (NH), 3089, 3028 (CH), 1585 (C=C) ; ^1^H-NMR (300 MHz, DMSO-*d*_6_): δ = 2.49 (s, 3H, CH_3_), 2.58 (s, 3H, CH_3_), 2.65 (s, 3H, CH_3_), 3.30 (s, 3H, CH_3_), 7.34–7.54 (m, 8H, ArH’s) and 10.65 (s, br, 1H, NH). Anal. Calcd. For C_22_H_21_N_8_S (464.99), C, 56.83; H, 4.55; N, 24.10; S, 6.90 Found C, 56.89; H, 4.60; N, 24.15; S, 6.85.

**Method B**: Benzenediazonium chloride (5 mmol), prepared in the usual way from aniline (0.46 g, 5 mmol), hydrochloric acid (1.5 mL, 6 M) and sodium nitrite (0.35 g, 5 mmol), was added dropwise with stirring to a cold solution of 2-(2-(1-(5-methyl-1-(*p*-tolyl)-1*H*-1,2,3-triazol-4-yl)ethylidene)hydrazinyl)-4-phenylthiazole (**12**) (1.9 g, 5 mmol) and sodium acetate (1.3 g, 10 mmol) in ethanol (30 mL) at 0–5 °C. The reaction mixture was stirred for 3 h in an ice bath and was left in refrigerator overnight. The solid was collect and crystallized from ethanol, giving a product identical (m.p., mixed mp and spectra) with **11b**.

### 3.4. 2-(2-(1-(5-Methyl-1-(p-tolyl)-1H-1,2,3-triazol-4-yl)ethylidene)hydrazinyl)-4-phenylthiazole (***13***)

**Method A**: A mixture of **9** (1.4 g, 5 mmol) and ω-bromoacetophenone (1 g, 5 mmol) in ethanol was refluxed for 4 h. The resulting solid that was collected and crystallized from ethanol gave a white crystal of **13**, Yield: 75%, m.p. 290 °C (Lit. m.p. 273 °C [22]). 

**Method B**: A mixture of 2-hydrazinyl-4-phenylthiazole (**12**) (1.76 g, 10 mmol), **1 **(2.1 g, 5 mmol) in ethanol (20 mL) and conc. hydrochloric acid (2 drops) was heated under reflux for 15 min. The solid was collected and crystallized from ethanol giving a product identical in all aspects (m.p., mixed m.p., and spectra) with the above sample obtained by **Method A**.

### 3.5. (E)-5-(2-Arylhydrazono)-2-((Z)-2-(1-(5-methyl-1-(p-tolyl)-1H-1,2,3-triazol-4-yl)ethylidene)-hydrazinyl)thiazol-4(5H)-one ***14a***–***c***

**Method A:** A mixture of **9** (1.4 g, 5 mmol), hydrazonoyl halide **3a** (5 mmol) and triethylamine (0.5 g, 0.7 ml, 5 mmol) in ethanol was boiled under reflux for 3 h. The resulting solid was collected and recrystallized from acetic acid afforded by **14a**

**Method B: **Dropwise addition of arenediazonium chlorides (5 mmol), which was prepared via reaction of the appropriate aniline, p-toluidine, p-chloroaniline (5 mmol), hydrochloric acid (1.5 mL, 6 M), sodium nitrite (0.37 g, 5 mmol) at 0–5 °C, to a mixture of **15** (1.64 g, 5 mmol) and sodium acetate (0.66 g, 5 mmol) in ethanol at 0–5 °C, while stirring. The reaction mixture was stirred for 3 h. The resulting solid was collected, washed with water and crystallized, giving **14a**–**c**. 

*(E)-2-((Z)-2-(1-(5-Methyl-1-(p-tolyl)-1H-1,2,3-triazol-4-yl)ethylidene)hydrazinyl)-5-(2-phenylhydrazono)thiazol-4(5H)-one* (**14a**). Yellow crystals from acetic acid, Yield 75%, m.p. 298–300 °C; FT-IR (KBr, cm^−1^): 3431, 3211 (2NH), 3051, 2920 (CH), 1581(C=C), 1659 (CO), 1604 (C=N); ^1^H-NMR (300 MHz, DMSO-*d*_6_): δ = 2.44 (s, 3H, CH_3_), 2.49 (s, 3H, CH_3_), 3.32 (s, 3H, CH_3_), 6.92–7.85 (m, 9H, ArH’s) 10.5 (s, br, 1H, NH) and 11.9 (s, br, 1H, NH). ^13^C-NMR (DMSO-*d*_6_) δ = 8.1, 19.2, 21.0, 115.4, 122.0, 123.8, 127.7, 129.8, 130.1, 139.2, 139.8, 145.7, 146.3, 147.2, 159.4, 167.9, 176.1. Anal. Calcd. For C_21_H_20_N_8_SO (432.51), C, 58.32; H, 4.66; N, 25.91; S, 7.41 Found C, 58.30; H, 4.69; N, 25.80; S, 7.50. 

*(E)-2-((Z)-2-(1-(5-Methyl-1-(p-tolyl)-1H-1,2,3-triazol-4-yl)ethylidene)hydrazinyl)-5-(2-(p-tolyl)hydrazono)thiazol-4(5H)-one* (**14b**). Brown crystals from ethanol, Yield: 80%, m.p. >300 °C; FT-IR (KBr, cm^−1^): 3437, 3267 (2NH), 2931 (CH), 1732 (CO), 1627 ν(C=N), 1573 ν(C=C). ^1^H-NMR (300 MHz, DMSO-*d*_6_): δ = 2.26 (s, 3H, CH_3_), 2.47 (s, 3H, CH_3_), 2.49(s, 3H, CH_3_), 2.53 (s, 3H, CH_3_), 7.41–7.50 (m, 8H, ArH’s), 8.41 (s, br, 1H, NH) and 10.9 (s, br, 1H, NH). ^13^C-NMR (DMSO-*d*_6_) δ = 8.1, 19.2, 20.6, 21.0, 117.4, 123.9, 128.6, 129.9, 130.2, 136.8, 139.2, 139.8, 145.2, 145.8, 146.3, 159.6, 167.9, 176.0. Anal. Calcd. For C_22_H_22_N_8_OS (446.54), C, 59.18; H, 4.97; N, 25.09; S, 7.18 Found C, 59.28; H, 4.89; N, 25.11; S, 7.28. 

*(E)-5-(2-(4-Chlorophenyl)hydrazono)-2-((Z)-2-(1-(5-methyl-1-(p-tolyl)-1H-1,2,3-triazol-4-yl)ethylidene)hydrazinyl)thiazol-4(5H)-one *(**14c**). Pale brown crystals from ethanol, Yield: 70%, m.p.: 263–265 °C; FT-IR (KBr, cm^−1^): 3431,3108 (2NH), 2972 (CH), 1664 (CO), 1634 (C=N), ^1^H-NMR (300 MHz, DMSO-*d*_6_): δ = 2.42 (s, 3H, CH_3_), 2.53 (s, 3H, CH_3_), 3.30 (s, 3H, CH_3_), 7.42–7.51 (m, 8H, ArH’s), 8.32 (s, br, 1H, NH) and 11.99 (s, br, 1H, NH). Anal. Calcd. For C_21_H_19_N_8_OSCl (466.96), C, 54.02; H, 4.10; N, 24.00; S, 6.87; Cl, 7.59 Found: C, 54.12; H, 4.20; N, 24.05; S, 6.90.

### 3.6. (*E*)-2-(2-(1-(5-Methyl-1-(*p*-tolyl)-1*H*-1,2,3-triazol-4-yl)ethylidene)hydrazinyl)thiazol-4(5*H*)-one (***15***)

A mixture of **9** (1.4 g, 5 mmol) and ethyl chloroacetate (0.61 g, 5 mmol) in ethanol was refluxed for 4 h. The resulting solid was collected and recrystallized from ethanol that gave white crystals of 1**5**, Yield: 75%, m.p. 255 °C. FT-IR (KBr, cm^−1^): 3116 (NH), 2951 (CH), 1735 (CO), 1624 (C=N). ^1^H-NMR (300 MHz, DMSO-*d*_6_): δ = 2.46 (s, 3H, CH_3_), 2.54 (s, 3H, CH_3_), 3.30 (s, 3H, CH_3_), 3.87 (s, 2H, OCH_2_), 7.42–8.32 (m, 4H, ArH’s) and 11.97 (s, br, 1H, NH). ^13^C-NMR (DMSO-*d*_6_) δ = 8.1, 19.1, 20.8, 37.1, 123.9, 129.7, 129.9, 139.2, 139.7, 146.0, 159.7, 168.0, 183.5. Anal. Calcd. For C_15_H_16_N_6_OS (328.40), C, 54.86; H, 4.91; N, 25.59; S, 9.76 Found: C, 54.90; H, 4.95; N, 25.34; S, 9.70. 

### 3.7. (*E*)-5-Benzylidene-2-((*E*)-2-(1-(5-methyl-1-(*p*-tolyl)-1*H*-1,2,3-triazol-4-yl)ethylidene)-hydrazinyl)thiazol-4(5*H*)-one (***16***)

A mixture of **15** (1.6 g, 5 mmol) and benzaldehyde (0.53 g, 5 mmol) in ethanol and catalytic amount of piperidine (5 drops) was refluxed for 3 h. The resulting solid was collected and recrystallized from acetic acid affording white crystals of **16**, Yield: 80%, m.p.: 283 °C. FT-IR (KBr, cm^−1^): 3124 (NH), 2974 (CH), 1705 (CO), 1624 (C=N). ^1^H-NMR (300 MHz, DMSO-*d*_6_): δ = 2.49 (s, 3H, CH_3_), 2.54 (s, 3H, CH_3_), 3.30 (s, 3H, CH_3_), 7.42–7.51 (m, 10H, ArH’s and = CH) and 11.91 (s, br, 1H, NH). ^13^C-NMR (DMSO-*d*_6_) δ = 8.1, 19.1, 20.8, 123.9, 129.2, 129.7, 129.9, 130.7, 132.5, 138.2, 139.3, 139.6, 146.2, 159.8, 167.8, 174.1. Anal. Calcd. For C_22_H_20_N_6_SO (416.51), C, 63.44; H, 4.84; N, 20.18; S, 7.70 Found: C, 63.50; H, 4.90; N, 20.20; S, 7.75.

### 3.8. 5-(Aryldiazenyl)-4-substituted-2-(3-(5-methyl-1-(p-tolyl)-1H-1,2,3-triazol-4-yl)-5-phenyl-4,5-dihydro-1H-pyrazole-1-yl)thiazole ***20a***–***d***, ***21***

**Method A**: A mixture of **17** (1.9 g, 5 mmol), the appropriate hydrazonoyl halides **3a**,**c**,**e**,**f or 3b** (5 mmol) and triethyl amine (0.5 mg, 0.75 mL, 5 mmol) in ethanol (20 mL) was heated under reflux for 4 h. The resulting solid was collected and recrystallized, giving thiazole derivatives **20a**–**d **and** 21**.

**Method B: **Benzenediazonium chloride (5 mmol) which was prepared via reaction of aniline (0.55 g, 5mmol), hydrochloric acid (3 mL, 6 M), and sodium nitrite (0.35 g, 5 mmol) was added dropwise, with stirring, to a cold solution of **21**. The reaction mixture was stirred for 3 h .The resulting solid was collected, washed with water and crystallized from ethanol, giving **20b**. 

*(E)-4-Methyl-2-(3-(5-methyl-1-(p-tolyl)-1H-1,2,3-triazol-4-yl)-5-phenyl-4,5-dihydro-1H-pyrazol-1-yl)-5-(phenyldiazenyl)thiazole* (**20a**). Orange crystals from acetic acid, Yield: 75%, m.p.: 235 °C; FT-IR (KBr, cm^−1^): 3045, 2926, 2860 (CH), 1653 (C=N), 1587 (C=C); ^1^H-NMR** (**300 MHz, DMSO-*d*_6_): δ = 2.49 (s, 3H, CH_3_), 2.56 (s, 3H, CH_3_), 2.70 (s, 3H, CH_3_), 3.72–3.74 (dd, 1H, H_b_), 3.78 (dd, 1H, H_a_), 5.78 (dd, 1H, H_x_) and 7.26–7.76 (m, 14H, ArH’s). ^13^C-NMR (DMSO-*d*_6_) δ = 8.1, 12.82, 21.0, 33.2, 63.6, 114.8, 121.7, 123.8, 127.5, 129.1, 129.5, 129.8, 130.1, 130.7, 130.9, 139.2, 139.7, 144.8, 149.6, 154.1, 161.1. MS (El, *m*/*z *(%)): 518 (M^+^, 100), 489 (5), 413 (2), 273 (15), 184 (20), 170 (15), 144 (25), 91 (35%), 77 (70), 65 (17). Anal. Calcd. For C_29_H_26_N_8_S (518.65), C, 67.16; H, 5.05; N, 21.61; S, 6.18 Found C, 67.26; H, 5.10; N, 21.69; S, 6.28.

*(E)-2-(3-(5-Methyl-1-(p-tolyl)-1H-1,2,3-triazol-4-yl)-5-phenyl-4,5-dihydro-1H-pyrazol-1-yl)-4-phenyl-5-(phenyldiazenyl)thiazole* (**20b**). Red crystals from acetic acid, Yield: 70%, m.p.: 275 °C; FT-IR (KBr, cm^−1^): 3043, 2924 (CH), 1666 (C=N); ^1^H-NMR (300 MHz, CDCl_3_): δ = 2.49 (s, 3H, CH_3_), 2.71 (s, 3H, CH_3_), 3.73–3.78 (dd, 1H, H_b_), 4.18 (dd, 1H, H_a_) 5.82 (dd, 1H, H_x_), and 7.33–8.18 (m, 19H, ArH^’^s),^ 13^C-NMR (DMSO-*d*_6_) δ = 8.1, 12.82, 21.0, 33.2, 63.6, 108.7, 121.7, 123.8, 126.7, 127.4, 128.2, 128.7, 128.2, 129.8, 129.9, 130.1, 130.4, 130.9, 133.1, 136.2, 139.1, 139.7, 144.7, 154.7, 168.0 MS (El, *m*/*z *(%): 580 (M^+^, 85), 551 (30), 447 (10), 367 (30), 133 (40), 91(50), 77(100), 65(20). Anal. Calcd. For C_34_H_28_N_8_S (580.72), C, 70.32; H, 4.86; N, 19.30; S, 5.52 Found C, 70.22; H, 4.75; N, 19.20; S, 5.56.

*(E)-4-Methyl-2-(3-(5-methyl-1-(p-tolyl)-1H-1,2,3-triazol-4-yl)-5-phenyl-4,5-dihydro-1H-pyrazol-1-yl)-5-(p-tolyldiazenyl)thiazole* (**20c**). Red crystals from acetic acid, Yield: 70%, m.p.: 240 °C; FT-IR (KBr, cm^−1^): 3039, 2926 (CH), 1658 (C=N); ^1^H-NMR (300 MHz, CDCl_3_): δ = 2.40 (s, 3H, CH_3_), 2.46 (s, 3H, CH_3_), 2.60 (s, 3H, CH_3_), 2.72 (s, 3H, CH_3_), 3.5–3.6 (dd, 1H, H_b_), 4.1–4.2 (dd, 1H, H_a_), 5.61–5.64 (dd, 1H, H_x_) and 6.81–8.17 (m, 13H, ArH’s). Anal. Calcd. For C_30_H_28_N_8_S (532.68), C, 67.65; H, 5.30; N, 2.04; S, 6.02 Found C, 67.72; H, 5.34; N, 2.14; S, 6.12.

*(E)-5-((4-Chlorophenyl)diazenyl)-4-methyl-2-(3-(5-methyl-1-(p-tolyl)-1H-1,2,3-triazol-4-yl)-5-phenyl-4,5-dihydro-1H-pyrazol-1-yl)thiazole* (**20d**). Red crystals from ethanol, Yield:65%, m.p.: 220 °C; FT-IR (KBr, cm^−1^): 3037, 2926 (CH), 1670 (C=N); ^1^H-NMR (300 MHz, CDCl_3_): δ = 2.48 (s, 3H, CH_3_), 2.56 (s, 3H, CH_3_), 2.69 (s, 3H,CH_3_), 3.63–3.72 (dd, 1H, H_b_), 4.11–4.21 (dd, 1H, H_a_), 5.64–5.70 (dd, 1H, H_x_) and 6.81–7.71 (m, 13H, ArH’s). Anal. Calcd. For C_29_H_25_N_8_SCl (553.09), C, 62.98; H, 4.56; N, 20.26; S, 5.80; Cl, 6.41 Found C, 62.85; H, 4.55; N, 20.30; S, 5.89.

### 3.9. 2-(3-(5-Methyl-1-(p-tolyl)-1H-1,2,3-triazol-4-yl)-5-phenyl-4,5-dihydro-1H-pyrazol-1-yl)-4-phenylthiazole (***21***)

A mixture of **17** (1.85 g, 5 mmol) and ω-bromoaceophenone (1 g, 5 mmol) in ethanol was refluxed for 4 h. The resulting solid was collected and crystallized from ethanol giving white crystals of **21**, Yield: 75%, m.p. 220 °C (Lit. m.p. 193 °C [25]).

### 3.10. 2-(3-(5-Methyl-1-(p-tolyl)-1H-1,2,3-triazol-4-yl)5–phenyl-4,5-dihydro-1H-pyrazole-1-yl)-5-(2-phenyl-hydrazono)thiazol-4(5H)-one (***22***)

A mixture of 4,5-dihydro-3-(5-methyl-1-(*p*-tolyl)-1*H*-1,2,3-triazol-4-yl)-5-phenyl-pyrazol-1-carbothioamide (**17**) (1.9 g, 5 mmol) and ethyl 2-chloro-2-(2-phenylhydrazono)acetate (**3b**) (1.1 g, 5 mmol) in ethanol (20 mL) was heated under reflux for 3 h. The resulting solid was collected and recrystallized from acetic acid, giving **22** as pale orange crystals. Yield 75%, m.p. 294–296 °C, FT-IR (KBr, cm^−1^): 3437 (NH), 3049, 2929 (CH), 1695 (CO), 1658 (C=N). ^1^H-NMR (300 MHz, CDCl_3_): δ = 2.48 (s, 3H, CH_3_), 2.65 (s, 3H, CH_3_), 3.73–3.85 (dd, 1H, H_b_), 4.09–4.19 (dd, 1H, H_a_), 5.77–5.81 (dd, 1H, H_x_), 7.26–7.38 (m, 15H, ArH’s and NH). ^13^C-NMR (DMSO-*d*_6_) δ = 8.1, 12.82, 21.0, 33.2, 67.2, 115.3, 122.3, 123.9, 127.0, 127.8, 128.7, 129.8, 129.9, 130.7, 139.1, 139.7, 144.0, 144.6, 146.7, 149.5, 156.6, 175.1. Anal. Calcd. For C_28_H_24_N_8_OS (520.62): C, 64.60; H, 4.65; N, 21.52; S, 6.16. Found, C, 64.67; H, 4.70; N, 21.60; S, 6.19.

### 3.11. 6-(5-Methyl-1-(*p*-tolyl)-1*H*-1,2,3-triazol-4-yl)-4-phenylpyridine derivatives (***24***)–(***29***)

General procedure: A mixture of **23** (1.5 g, 5 mmol), the appropriate acetylacetone, ethyl acetoacetate, ethyl cyanoacetate, cyanothioacetamide, malononitrile, benzoylacetonitrile and ammonium acetate (0.38 g, 5 mmol) in acetic acid (10 mL) was heated under reflux for 4 h. The resulting solid was filtered, washed with water and crystallized from the proper solvent, giving pyridine derivatives **24**–**29**.

*Ethyl 2-methyl-6-(5-methyl-1-(p-tolyl)-1H-1,2,3-triazol-4-yl)-4-phenylnicotinate* (**24**). Yellow crystals from ethanol, yield 85% m.p.: 195 °C, FT-IR (KBr, cm^−1^): 3035, 2953 (CH); 1660 (CO), 1629 (C=N); 1579 (C=C); ^1^H-NMR (300 MHz, CDCl_3_): δ = 1.34 (t, 3H, CH_2_**CH_3_**), 2.42 (s, 3H, CH_3_), 2.60 (s, 3H, CH_3_), 2.69 (s, 3H, CH_3_), 4.20 (q, 2H, **CH_2_**CH_3_), 7.27–7.73 (m, 10H, ArH’s and pyridine H-5). ^13^C-NMR (DMSO-*d*_6_) δ = 11.5, 13.82, 20.9, 23.5, 61.7, 63.6, 93.3, 118.6, 123.0, 125.3, 125.8, 129.4, 129.8, 133.6, 134.52, 142.4, 146.0, 154.3, 167.9, 175. MS (El, *m*/*z *(%): 412 (M^+^, 15), 395 (10), 384 (50), 325 (30), 342 (10), 247 (70), 132 (100), 103 (80), 91 (90), 77 (40), 65 (55). Anal. Calcd. for C_25_H_24_O_2_N_4_ (412.49), C, 72.80; H, 5.86; N,13.60. Found: C, 72.86; H, 5.90; N, 13.65. 

*1-(2-Methyl-6-(5-methyl-1-(p-tolyl)-1H-1,2,3-triazol-4-yl)-4-phenylpyridin-3-yl)ethan-1-one* (**25**). Orange crystals from acetic, yield 70% m.p.: 190 °C, FT-IR (KBr, cm^−1^): 3002, 2949 (CH); 1737 (CO); 1614 (C=N); 1579 (C=C). ^1^H-NMR (300 MHz, CDCl_3_): δ = 2.09 (s, 3H, CH_3_), 2.43 (s, 3H, CH_3_), 2.46 (s, 3H, CH_3_), 2.66 (s, 3H, CH_3_) and 7.26–8.14 (m, 10H, ArH^’^s and pyridine H-5), MS (El, *m*/*z *(%): 384 (M^+2^, 20), 369 (10), 354 (60), 341 (70), 247 (40), 194 (35), 144 (15), 132 (95), 91 (99), 77 (40), 65 (60). Anal. Calcd. For C_24_H_22_ON_4_, (382.47): C, 75.37; H, 5.80; N, 14.65. Found C, 75.47; H, 5.95; N, 14.75.

*2-Amino-6-(5-methyl-1-(p-tolyl)-1H-1,2,3-triazol-4-yl)4–phenylpyridine-3-carbonitrile* (**26**). Yellow crystals from acetic acid, Yield 75%, m.p.: 197 °C, FT-IR (KBr, cm^−1^): 3427, 3224 ν(NH_2_); 3002, 2954 ν(CH); 2276 ν(CN); 1635 ν(C=N); 1581 ν(C=C), ^1^H-NMR ( 300 MHz, CDCl_3_): δ = 2.46 (s, 3H, CH_3_), 2.65 (s, 3H, CH_3_), 6.95 (s, br, 2H, NH_2_), 7.26–8.14 (m, 10H, ArH’s and pyridineH-5)*. *^13^C-NMR (DMSO-*d*_6_) δ = 11.5, 20.9, 63.6, 91.7, 97.8, 118.4, 118.8, 121.3, 127.5, 128.8, 129.7, 133.52, 133.7, 140.3, 142.5, 150.6, 160.7, 166. MS (EI, *m*/*z *(%)): 366 (M^+^, 60), 351 (30), 338 (40), 247 (50), 194 (0), 144 (30), 132 (70), 103 (50), 91 (70), 80 (100), 64 (50). Anal. Calcd. for C_22_H_18_N_6 _(366.43), C,72.11; H, 4.95; N, 22.94 Found: C, 72.15; H, 4.85; N, 22.88.

*6-(5-Methyl-1-(p-tolyl)-1H-1,2,3-triazol-4-yl)-2-oxo-4-phenyl-pyridine-3-carbonitrile* (**27**). Yellow crystals from acetic acid. Yield 75%, m.p. 193 °C, FT-IR (KBr, cm ^−1^): 3433 (NH), 3043, 2929 (CH); 1668 (CO), 1643 (C=N), 1581 (C=C); ^1^H-NMR (300 MHz, CDCl_3_): δ = 2.47 (s, 3H, CH_3_), 2.65 (s, 3H, CH_3_), 7.26–8.14 (m, 10H, ArH’s and pyridine H-5), 11.65 (s, br, 1H, NH), MS (El, *m*/*z *(%)): 368 (M^+1^, 40), 304 (10), 247 (65), 194 (55), 132 (100), 115 (30), 103 (70), 91 (85), 77 (40), 65 (50). Anal. Calcd. For C_22_H_17_N_5_O (367.41), C, 71.92; H, 4.66; N, 19.06 Found: C, 71.89; H, 4.65; N, 19.16.

*6-(5-Methy-1-(p-tolyl)-1H-1,2,3-triazol-4-yl)-4-phenyl-2-thioxo-pyridine-3-carbonitrile* (**28**). Orange crystals from acetic acid, Yield 70%, m.p. 278 °C, FT-IR (KBr, cm^−1^): 3437 (NH); 3037, 2920 (CH); 2211 (CN), 1584 (C=C), 1615 (C=N), ^1^H-NMR (300 MHz, CDCl_3_): δ = 2.41 (s, 3H, CH_3_), 2.49 (s, 3H, CH_3_), 4.47–8.12 (m, 10H, ArH’s, pyridine H-5), 15.45 (S, br, 1H, NH). ^13^C-NMR (DMSO-*d*_6_) δ = 8.5, 20.9, 108.4, 112.2, 118.3, 123.2, 128.8, 129.7, 129.9, 130.4, 135.4, 135.8, 137.4, 139.4, 139.8, 150.3, 150.7, 177.8. MS (El, *m*/*z *(%)): 383 (M^+^, 40), 303 (5), 247 (50), 194 (50), 132 (100), 103 (60), 90 (100), 77 (50), 68 (60). Anal. Calcd. For C_22_H_17_N_5_S (383.48), C, 68.91; H, 4.47; N, 18.26; S, 8.36. Found C, 68.89; H, 4.37; N, 18.30 S, 8.46.

*(2-Amino-6-(5-methyl-1-(p-tolyl)-1H-1,2,3-triazol-4-yl)-4-phenylpyridin-3-yl)(phenyl)-methanone *(**29**). Pale yellow crystals from ethanol, Yield 70%, m.p.: 183 °C, FT-IR (KBr, cm^−1^): 3431, 3330 (NH_2_); 2972, 2925 (CH), 1659 (CO); 1596 (C=C). ^1^H-NMR (300 MHz, CDCl_3_): δ = 2.49 (s, 3H, CH_3_), 2.56 (s, 3H, CH_3_), 6.93 (s, br, 2H, NH_2_), 7.26–8.12 (m, 15H, ArH’s and pyridine H-5). Anal. Calcd. For C_28_H_23_N_5_O (445.53), C, 75.49; H, 5.20; N, 15.72 Found C, 75.39; H, 5.40; N, 15.65. 

### 3.12. 2-Methyl-6-(5-methyl-1-(*p*-tolyl)-1*H*-1,2,3-triazol-4-yl)-4-phenylnicotinohydrazide (***31***)

Equimolar amounts of ethyl 6-(5-methyl-1-(*p*-tolyl)-1*H*-1,2,3-triazol-4-yl)-4-phenyl pyridine-3-carboxylate (**24**) (2.2 g, 5 mmol) and hydrazine hydrate (1 mL, 10 mmol) in ethanol (10 mL) were refluxed for 5 h. The resulting solid was collected and recrystallized, giving **31** as white crystals from ethanol, Yield 89%, m.p. 145 °C, FT-IR (KBr, cm^−1^): 3431, 3335 (NH_2_); 2960, 2923 (CH); 1662 (CO); 1572 (C=C). ^1^H-NMR (300 MHz, DMSO-*d*_6_): δ = 2.24 (s, br, 2H, NH_2_), 2.42 (s, 3H, CH_3_), 2.60 (s, 3H, CH_3_), 2.97 (s, 3H, CH_3_), 10.20 (s, br, 1H, NH), 7.11–7.61 (m, 10H, ArH’s and pyridine H-5). Anal. Calcd. For C_23_H_22_·N_6_O (398.42): C, 69.33; H, 5.57; N, 21.09 Found C, 69.35; H, 5.60; N, 21.19.

### 3.13. (3,5-Dimethyl-1H-pyrazol-1-yl)(2-methyl-6-(5-methyl-1-(p-tolyl)-1H-1,2,3-triazol-4-yl)-4-phenyl-pyridin-3-yl)methanone (***32***) and 5-Methyl-2-(2-methyl-6-(5-methyl-1-(p-tolyl)-1H-1,2,3-triazol-4-yl)-4-phenyl-pyridine-3-carbonyl)-2,4-dihydropyrazol-3-one (***33***)

Equimolar amounts of **31** and the appropriate acetylacetone or ethyl acetoacetate (4 mmol for each) in ethanol (10 mL), with two drops of acetic acid, were refluxed for 4 h. The resulting solid was collected and recrystallized from ethanol, giving the corresponding products **32 **and** 33**, respectively.

*(3,5-Dimethyl-1H-pyrazol-1-yl)(2-methyl-6-(5-methyl-1-(p-tolyl)-1H-1,2,3-triazol-4-yl)-4-phenyl-pyridin-3-yl) methanone* (**32**). White crystals from ethanol, Yield 80%, m.p. 207 °C, FT-IR (KBr, cm^−1^): 3032, 2961, 2941, 2839 (CH); 1641 (CO); 1589 (C=C). ^1^H-NMR (300 MHz, DMSO-*d*_6_): δ = 2.41 (s, 3H, CH_3_) 2.48 (s, 3H, CH_3_), 2.49 (s, 3H, CH_3_), 2.57 (s, 3H, CH_3_), 2.70 (s, 3H, CH_3_), 7.21–7.57 (m, 11H, ArH’s, pyridine H-5 and pyrazole H-4). Anal. Calcd. For C_28_H_26_·N_6_O (462.56): C, 72.71; H, 5.67; N, 18.17 Found C, 72.80; H, 5.81; N, 18.27.

*5-Methyl-2-(2-methyl-6-(5-methyl-1-(p-tolyl)-1H-1,2,3-triazol-4-yl)-4-phenyl-pyridine-3-carbonyl)-2,4-dihydropyrazol-3-one* (**33**). White crystals from ethanol, Yield 80%, m.p. 217 °C, FT-IR (KBr, cm^−1^): 3434 (OH); 2976, 2925 ν(CH); 1682 (CO); 1609 (C=N), 1575 (C=C). ^1^H-NMR (300 MHz, DMSO-*d*_6_): δ = 2.21 (s, 3H, CH_3_) 2.48 (s, 3H, CH_3_), 2.57 (s, 3H, CH_3_), 2.62 (s, 3H, CH_3_), 4.67 (s, 2H, pyrazoline H-4), 7.19–7.52 (m, 10H, ArH’s, pyridine H-5). Anal. Calcd. For C_27_H_24_N_6_O_2_ (464.53): C, 69.81; H, 5.21; N, 18.09 Found C, 69.91; H, 5.33; N, 18.19

### 3.14. Azido (2-Methyl-6-(5-methyl-1-(p-tolyl)-1H-1,2,3-triazol-4-yl)-4-phenyl-pyridin-3-yl)-methanone (***34***)

To a stirred solution of **31 **(5 mmol) in acetic acid (15 mL) at 0–5 °C, sodium nitrite was added portion-wise until effervescence ended. The reaction mixture was stirred for 1 h. The resulting solid was collected, filtered, washed with water and recrystallized, giving the azido derivative **34**. Buff crystals from acetic acid, yield (86%) m.p. 160 °C, FT-IR (KBr, cm^−1^): 2964, 2924 (CH); 1641 (CO), 1609 (C=C). ^1^H-NMR (300 MHz, DMSO-*d*_6_): δ = 2.44 (s, 3H, CH_3_), 2.50 (s, 3H, CH_3_), 2.69 (s, 3H, CH_3_), 7.17–7.57 (m, 10H, ArH’s and pyridine H-5). Anal. Calcd. For C_23_H_19_N_7_O (409.49): C, 67.47; H, 4.68; N, 23.95 Found C, 67.50; H, 4.70; N, 23.99.

### 3.15. 4-(Aryldiazenyl-3,5-dimethylpyrazol-1-yl)(2-methyl-6-(5-methyl-1-(p-tolyl)-1H-1,2,3-triazol-4-yl)-4-phenyl-pyridin-3-yl]methanone (***35a***, ***35b***) and 4-(Arylyhydrazono)-5-methyl-2(2-methyl-6-(5-methyl-1-(p-tolyl)-1H-1,2,3-triazol-4-yl)-4-phenyl-pyridin-3-carbonyl)-2,4-dihydropyrazol-3-one (***36a***, ***36b***)

Dropwise addition of the appropriate arenediazonium chloride (5 mmol), which was prepared via reaction of appropriate aniline or *p*-toluidine (5 mmol), hydrochloric acid (1.5 mL, 6M) and sodium nitrite (0.37 g, 5 mmol) at 0–5 °C, to a mixture of the appropriate **32** or** 33** (5 mmol) and sodium acetate (1.3 g, 5 mmol) in ethanol (30 mL) at 0–5 °C while stirring the reaction mixture was stirred for 3 h. The resulting solid was collected, washed with water and recrystallized from acetic acid, giving **35a**,** 35b**, **36a **and** 36b**, respectively.

*(E)-(3,5-Dimethyl-4-(phenyldiazenyl)-1H-pyrazol-1-yl)(2-methyl-6-(5-methyl-1-(p-tolyl)-1H-1,2,3-triazol-4-yl)-4-phenylpyridin-3-yl)methanone* (**35a**). Orange crystals from acetic acid, Yield 70%, m.p. 170 °C, FT-IR (KBr, cm^−1^): 2925 (CH); 1722 (CO); 1608 (C=N), 1566 (C=C): ^1^H-NMR (300 MHz, DMSO-*d*_6_): δ = 2.28 (s, 3H, CH_3_) 2.44 (s, 3H, CH_3_), 2.50 (s, 3H, CH_3_), 2.60 (s, 3H, CH_3_), 2.69 (s, 3H, CH_3_) and 7.17–7.97 (m, 15H, ArH’s, pyridine H-5). Anal. Calcd. For C_34_H_30_N_8_O (566.67): C, 72.07; H, 5.34; N, 19.77 Found C, 72.16; H, 5.29; N, 19.88

*(E)-(3,5-Dimethyl-4-(p-tolyldiazenyl)-1H-pyrazol-1-yl)(2-methyl-6-(5-methyl-1-(p-tolyl)-1H-1,2,3-triazol-4-yl)-4-phenylpyridin-3-yl)methanone* (**35b**). Orange crystals from acetic acid, Yield 70%,m.p. 175 °C, FT-IR (KBr, cm^−1^): 2966, 2924 (CH); 1722 (CO); 1647 (C=N); 1605 (C=C). ^1^H-NMR (300 MHz, DMSO-*d*_6_): δ = 2.25 (s, 3H, CH_3_) 2.44 (s, 3H, CH_3_), 2.49 (s, 3H, CH_3_), 2.50 (s, 3H, CH_3_), 2.69 (s, 3H, CH_3_), 2.71 (s, 3H, CH_3_) and 7.17–7.98 (m, 14H, ArH’s, pyridine H-5). Anal. Calcd. For C_35_H_32_N_8_O (580.70): C, 72.39; H, 5.55; N, 19.30 Found C, 72.49; H, 5.66; N, 19.40.

*(E)-5-Methyl-2-(2-methyl-6-(5-methyl-1-(p-tolyl)-1H-1,2,3-triazol-4-yl)-4-phenylnicotinoyl)-4-(phenyldiazenyl)-2,4-dihydro-3H-pyrazol-3-one* (**36a**). Orange crystals from acetic acid, Yield 70%, m.p. 165 °C, FT-IR (KBr, cm^−1^): 3432 (OH); 2975, 2921 (CH); 1721 (CO); 1679 (C=N); 1584 (C=C). ^1^H-NMR (300 MHz, DMSO-d_6_): δ = 2.31 (s, 3H, CH_3_) 2.43 (s, 3H, CH_3_), 2.58 (s, 3H, CH_3_), 2.69 (s, 3H, CH_3_), 4.35 (s, br, 1H, pyrazoline), 7.09–7.58 (m, 15H, ArH’s and pyridine H-5). ^13^C-NMR (DMSO-d_6_) δ = 9.6, 11.8, 20.8, 24.4, 115.3, 123.4, 125.7, 126.8, 128.4, 129.7, 130.4, 132.4, 133.3, 138.8, 139.5, 139.8, 140.8, 169.0, 171.2, 172.5, 170.0. Anal. Calcd. For C_33_H_28_N_8_O_2_ (568.64): C, 69.70; H, 4.96; N, 19.71 Found C, 69.65; H, 4.85; N, 19.72.

*(E)-5-Methyl-2-(2-methyl-6-(5-methyl-1-(p-tolyl)-1H-1,2,3-triazol-4-yl)-4-phenylnicotinoyl)-4-(p-tolyldiazenyl)-2,4-dihydro-3H-pyrazol-3-one* (**36b**). Orange crystals from acetic acid, Yield 70%, m.p. 170 °C, FT-IR (KBr, cm^−1^): 2974, 2922 (CH); 1721 (C=O); 1649 (C=N); 1608 (C=C). ^1^H-NMR (300 MHz, DMSO-*d*_6_): δ = 2.42 (s, 3H, CH_3_), 4.50 (s, 1H, pyrazoline), 2.49 (s, 3H, CH_3_), 2.57 (s, 3H, CH_3_), 2.69 (s, 3H, CH_3_), 2.71 (s, 3H, CH_3_), and 7.14–7.57 (m, 14H, ArH’s and pyridine H-5). Anal. Calcd. For C_34_H_30_N_8_O_2_ (582.67): C, 70.09; H, 5.19; N, 19.23 Found C, 70.19; H, 5.20; N, 19.10.

### 3.16. 1-(2-Methyl-6-(5-methyl-1-(p-tolyl)-1H-1,2,3-triazol-4-yl)-4-phenyl-pyridin-3-yl)-3-substituted urea (***38a***, ***38b***) and 3-(2-Methyl-6-(methyl-1-(p-tolyl)-1H-1,2,3-triazol-4-yl)-4-phenyl-pyridin-3-yl)quinazoline-2,4-(1H,3H)dione (***39***)

A mixture of **34** (2 g, 5mmol) and appropriate aniline, p-toluidine, anthranilic acid (or methyl anthranilate) (5 mmol) in dry dioxane (20 mL) was refluxed for 4 h. The resulting solid that was collected and recrystallized from the proper solvent gave **38a**, **38b **and** 39**, respectively

*1-(2-Methyl-6-(5-methyl-1-(p-tolyl)-1H-1,2,3-triazol-4-yl)-4-phenylpyridin-3-yl)-3-phenylurea* (**38a**). White crystals from acetic acid yield 70%, m.p. 180 °C. FT-IR (KBr, cm^−1^): 3426 (NH); 2983, 2926 (CH); 1722 (CO); 1647 (C=N); 1594 (C=C). ^1^H-NMR (300 MHz, DMSO-*d*_6_): δ = 2.43 (s, 3H, CH_3_) 2.59 (s, 3H, CH_3_), 2.71 (s, 3H, CH_3_), 7.44–7.97 (m, 15H, ArH’s and pyridine H-5), 8.88 (s, br, 2H, 2NH). Anal. Calcd. For C_29_H_26_N_6_O (474.57): C, 73.40; H, 5.52; N, 17.71 Found C, 73.37; H, 5. 63; N, 17.69.

*1-(2-Methyl-6-(5-methyl-1-(p-tolyl)-1H-1,2,3-triazol-4-yl)-4-phenylpyridin-3-yl)-3-p-tolylurea* (**38b**). White crystals from acetic acid yield 72%, m.p. 170–172 °C. FT-IR (KBr, cm^−1^): 3423 (NH); 2984, 2962, 2952 (CH); 1722 (CO); 1592 (C=C), ^1^H-NMR (300 MHz, DMSO-*d*_6_): δ = 2.44 (s, 3H, CH_3_) 2.49 (s, 3H, CH_3_), 2.60 (s, 3H, CH_3_), 2.71 (s, 3H, CH_3_), 7.27–7.98 (m, 14H, ArH’s and pyridine H-5), 8.90 (s, br, 2H, 2NH). Anal. Calcd. For C_30_H_28_N_6_O (488.60): C, 73.75; H, 5.17; N, 17.20 Found C, 73.80; H, 5.20; N, 17.30.

*3-(2-Methyl-6-(5-methyl-1-(p-tolyl)-1H-1,2,3-triazol-4-yl)-4-phenyl-pyridin-3-yl)-qninazoline-2,4-(1H,3H)dione* (**39**). White crystals from acetic acid, yield 65%, m.p. 190 °C. FT-IR (KBr, cm^−1^): 3424 (NH); 2983, 2926, 2875 (CH); 1722 (CO); 1594 (C=C). ^1^H-NMR (300 MHz, DMSO-*d*_6_): δ = 2.43 (s, 3H, CH_3_), 2.50 (s, 3H, CH_3_), 2.59 (s, 3H, CH_3_), 7.44–7.97 (m, 14H, ArH’s and pyridine H-5), 10.54 (s, br, 1H, NH), ^13^C-NMR (DMSO-*d*_6_) δ = 8.8, 20.5, 21.1, 114.2, 115.6, 117.2, 121.9, 123.7, 123.8, 128.5, 129.8, 132.7, 134.2, 134.6, 135.2, 137.8, 138.4, 138.7, 139.5, 140.2, 141.3. 144.2, 153.1, 158.6, 161.7, 164.6. Anal. Calcd. For C_30_H_24_N_6_O_2_ (500.56): C, 71.99; H, 4.83; N, 16.79 Found C, 71.89; H, 4.79; N, 16.85.

### 3.17. Phenyl 2-Methyl-6-(5-methyl-1-(p-tolyl)-1H-1,2,3-triazol-4-yl)-4-phenylpyridin-3-yl)-carbamate (***40***)

A mixture of **34** (2 g, 5 mmol) and phenol (0.47 g, 5 mmol) in dry benzene (20 mL) was refluxed for 4 h. The resulting solid was collected and crystallized from ethanol, affording the corresponding **40**, as buff crystals, yield 70%, m.p. 140–142 °C. FT-IR (KBr, cm^−1^): 3425 (NH); 2984, 2925, 2866 (CH); 1722 (CO); 1597 (C=C). ^1^H-NMR (300 MHz, DMSO-*d*_6_): δ = 2.44 (s, 3H, CH_3_) 2.49 (s, 3H, CH_3_), 2.69 (s, 3H, CH_3_), 7.33–7.97 (m, 15H, ArH’s and pyridine H-5), 11.65 (s, br, 1H, NH); Anal. Calcd. For C_29_H_25_N_5_O_2_ (475.55): C, 73.25; H, 5.30; N, 14.73; Found C, 73.35; H, 5.40; N, 14.85.

## 4. Conclusions

Compound **1** proved to be useful for synthesis of a new series of novel functionalized 1,3,4-thiadiazoles, 1,3-thiazoles and pyridines containing 1,2,3-triazole moiety using hydrazonoyl halides as precursors. Also, compound **31 **proved to be a useful precursor in the synthesis of various pyrazoles, urea and carbamate derivatives. The biological activities of the synthesized products will be reported in extended work.
